# Mn–Fe_3_O_4_ heterogeneous Fenton catalytic oxidation: mechanism and performance in sauce-flavored liquor wastewater degradation

**DOI:** 10.1039/d6ra00561f

**Published:** 2026-03-25

**Authors:** Benfu Luo, Jie Yu, Yujing Yan, Weiwei Huang, Jinyin Li, Yuhang Liu, Xi Yang, Xiang Zhou, Haiyan Ning

**Affiliations:** a School of Architecture and Civil Engineering, Xihua University Chengdu 610039 China kkllbbff@mail.xhu.edu.cn Y540751@163.com yooho666@outlook.com l17671290047@163.com petrel@mail.xhu.edu.cn +86 15828458283; b China Municipal Engineering Zhongnan Design and Research Institute Co., Ltd Wuhan 430010 China hww1010@foxmail.com ljy_1102@163.com; c Chengdu University Library Chengdu 610106 China xiyang151503@163.com; d Suyi Design Group Co., Ltd Nanjing 2100 China xuzhu719@126.com

## Abstract

This study aims to investigate the mechanism of synthesized Mn–Fe_3_O_4_ catalysts in the deep degradation of COD in sauce-flavored liquor wastewater by heterogeneous Fenton oxidation. Additionally, the study evaluates the impact of operational factors, including pH, catalyst composition, and dosage on the COD removal rate. The physicochemical characteristics of Mn–Fe_3_O_4_ catalysts were comprehensively analyzed using scanning electron microscopy (SEM), energy-dispersive X-ray spectroscopy (EDS), X-ray diffraction (XRD), and X-ray photoelectron spectroscopy (XPS). The oxidation mechanism of the Mn–Fe_3_O_4_ heterogeneous Fenton system was elucidated through gas chromatography–mass spectrometry (GC-MS) analysis, kinetic modeling, and radical quenching experiments involving *tert*-butanol (TBA) and benzoquinone (BQ). The results demonstrated that the Mn–Fe_3_O_4_-based system enhanced COD removal by 26% compared to the conventional Fenton process, exhibiting remarkable stability and magnetic recoverability. The catalyst system followed second-order kinetics, with the dual Mn–Fe active centers on the catalyst surface facilitating electron transfer *via* charge redistribution, promoting redox cycling of Fe^2+^/Fe^3+^ and Mn^2+^/Mn^3+^, and significantly increasing hydroxyl radical (·OH) production, thereby enabling the efficient degradation of refractory organic pollutants. This study provides valuable insights for the development of innovative catalytic materials for the effective treatment of industrial wastewater containing phenolic quinones, which are challenging to degrade, and sets the stage for further industrial applications.

## Abstract 2

This study aims to investigate the mechanism and performance of synthesized Mn–Fe_3_O_4_ catalysts in the deep degradation of COD in sauce-flavored liquor wastewater by heterogeneous Fenton oxidation.

## Graphical abstract

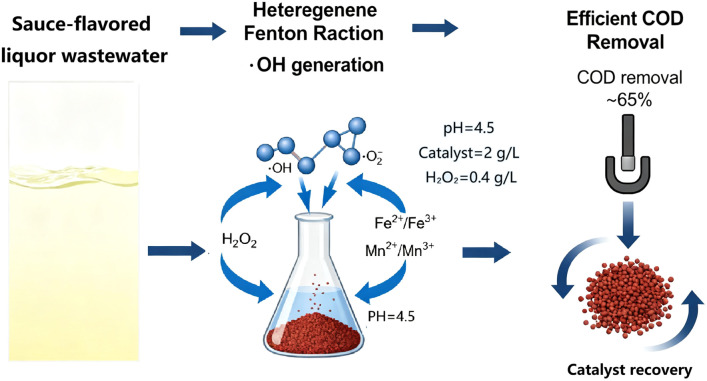

## 1. Introduction

The rapid expansion of sauce-flavored liquor production has increased wastewater discharge and raised growing environmental concerns. Sauce-flavored liquor wastewater contains large quantities of organic matter and suspended solids,^1,2^ accompanied by complex chemical constituents and acidic conditions, which make conventional treatment processes less effective.^3^ As a result of these combined characteristics, the wastewater exhibits poor treatability and presents considerable challenges for effective environmental management. To meet the increasingly stringent pollutant discharge standards enforced across various basins in China, the current treatment approach for sauce-flavored liquor wastewater typically follows a multi-stage process, comprising pre-treatment, anaerobic treatment, aerobic treatment, and advanced treatment. The advanced treatment unit typically employs secondary coagulation and precipitation, biologically aerated filters, carbon filtration, nanofiltration, ozone oxidation, Fenton oxidation,^4^ and other technologies. Although this approach can reduce pollutant concentrations in wastewater to some extent, its potential for further improvement in removal efficiency remains limited. Specifically, after the pre-treatment reduces the COD from an initial concentration of 20 000 mg L^−1^ to 80 mg L^−1^, further reduction to meet the discharge limit of 50 mg L^−1^ is challenging with conventional homogeneous Fenton and ozone oxidation processes. Additionally, these methods incur high treatment costs. Consequently, the development of efficient, cost-effective, and environmentally friendly advanced oxidation technologies is crucial for addressing the pollution issues associated with sauce-flavored liquor wastewater.

As a representative advanced oxidation technique. In the Fenton-based oxidation system, highly reactive radical species are produced, which are capable of rapidly oxidizing a wide range of organic compounds without selectivity.^5^ This method offers the advantages of mild reaction conditions and ease of operation. However, the traditional Fenton method has limitations such as a narrow pH application range, the generation of iron sludge, and the difficulty in recovering the catalyst,^6,7^ which affect its practical engineering application. The advent of heterogeneous Fenton technology addresses these challenges while preserving the high catalytic efficiency of the Fenton reaction. By immobilizing iron ions or other metal ions on solid-phase carriers or directly using solid metal oxides as catalysts, this method replaces the traditional free iron ions.^8^ This approach not only expands the pH range of applicability but also enables catalyst recycling and reuse, thereby reducing treatment costs. As a result, the use of heterogeneous Fenton oxidation with solid catalysts to degrade organic matter in sauce-flavored liquor wastewater has emerged as a valuable option.

Common heterogeneous Fenton oxidation catalysts available in the market are typically developed to address the advanced oxidation needs of industries such as printing and dyeing, and pharmaceuticals. However, these catalysts face significant limitations, including their ineffectiveness for the deep degradation of sauce-flavored liquor wastewater and their high costs. Fe_3_O_4_ (magnetite), a widely used magnetic material, has shown promise as a heterogeneous Fenton catalyst.^9^ The compositional modification and surface functionalization of ferromagnetic materials can significantly regulate their properties, thereby enhancing their interface reactions and multi-functional performance in environmental applications, such as catalytic oxidation processes.^10^ In this study, we leverage the magnetic recyclability of Fe_3_O_4_ to overcome the issue of catalyst loss typically associated with particulate catalysts. Furthermore, we enhance the catalytic properties by loading Mn as an active component onto the carrier using the impregnation method. The results indicate that, when considering factors such as catalytic efficiency, cost-effectiveness, and catalyst stability, the catalytic oxidation using the synthesized Mn–Fe_3_O_4_ catalyst is a more effective approach for degrading organic matter in sauce-flavored liquor wastewater. The COD removal rate achieved is approximately 65%, demonstrating its significant potential for practical application.

Numerous theoretical studies have confirmed the catalytic oxidation efficiency of iron-based catalysts. Gaber N. M. *et al.*^11^ developed a magnetic separable MnFe_2_O_4_/CoNiFe-LTH/g-C_3_N_4_ composite catalyst for activating H_2_O_2_ to degrade doxycycline. This catalyst demonstrated highly efficient catalytic activity and good stability under near-neutral conditions, achieving 89.11% degradation of the target pollutant within 60 minutes. Du J. *et al.*^12^ developed a magnetic porous Mn/Fe_3_O_4_ catalyst to activate peroxymonosulfate for the degradation of bisphenol A. The catalyst demonstrated excellent catalytic performance, significantly accelerating the efficient decomposition of bisphenol A. Zhou H. *et al.*^13^ prepared a Mn–Fe_3_O_4_ catalyst using the one-pot method. The results revealed that the catalyst facilitated the rapid degradation of ciprofloxacin across a wide pH range (pH = 4.5 to 9.5), greatly expanding the application conditions of Fenton oxidation. While the aforementioned studies validated catalytic efficiency under laboratory conditions, the heterogeneous catalytic behavior in actual sauce-flavored liquor wastewater, under the synergistic influence of multiple components, requires further systematic investigation.

The objective of this study was to investigate the COD degradation efficiency of synthesized Mn–Fe_3_O_4_ catalysts during the advanced treatment stage of sauce-flavored liquor wastewater, as well as to explore the underlying reaction mechanisms. The effects of varying pH values, catalyst types, and catalyst dosages on COD removal from sauce-flavored liquor wastewater *via* the heterogeneous Fenton reaction were examined. We explored the active components on the catalyst surface and the role of ·OH in the heterogeneous Fenton reaction with Mn–Fe_3_O_4_. Additionally, we evaluated the method of magnetic catalyst recycling, optimized experimental parameters, and established the optimal reaction conditions and kinetic equations. The study also elucidates the degradation mechanism of organic matter, offering a novel technological pathway for the efficient and in-depth treatment of sauce-flavored liquor wastewater.

## 2. Materials and methods

### 2.1 Chemicals and instruments

The main instrumentation in the test were digital display constant temperature magnetic stirrer (HJ-4), electric thermostatic drying oven (202-EBS), muffle furnace (SX-2-4-10A), HQ multi-parameter water quality analyzer (HQ1110), low-speed centrifuge (SN-LSC-3), ultra pure water device (UPT-I-5/10/20T), pH meter (PHS-25), electronic balance (BSA224S), standard COD digester (HCA-100), fully automatic specific surface area and porosity analyzer (ASAP 2460), scanning electron microscope (ZEISS Gemini SEM 300), X-ray photoelectron spectrometer (K-Alpha), gas chromatography-mass spectrometry (GCMS-QP2020NX).

The pharmaceuticals used in this test were of analytical grade. The medicines are H_2_SO_4_, HCl, HgSO_4_, Ag_2_SO_4_, K_2_Cr_2_O_7_, NaOH, (NH_4_)_2_Fe(SO_4_)_2_·6H_2_O, 30% H_2_O_2_, 7.5 wt% H_2_O_2_ (diluted from 30 wt% hydrogen peroxide stock solution), NaHSO_3_, FeSO_4_, C_6_H_4_O_2_.

### 2.2 Experimental methodology

#### 2.2.1 Experimental water

The test water was sourced from the effluent of the secondary biochemical sedimentation tank at the Wujiagou Sewage Treatment Plant, operated by the Langjiu Group, prior to its passage through the advanced treatment unit. The sample was odorless, contained only trace amounts of flocculent matter, and exhibited a pale yellowish-brown hue.

#### 2.2.2 Preparation of catalyst

In this experiment, a loaded Fe_3_O_4_ heterogeneous catalyst was prepared using the impregnation method. The preparation process was divided into three steps, as follows:

Step 1: Fe_3_O_4_ was used as the carrier, with Mn(NO_3_)_2_, Fe(NO_3_)_3_·9H_2_O, and Cu(NO_3_)_2_·3H_2_O serving as the raw materials. The Fe_3_O_4_ was thoroughly washed with ultrapure water, and filtration was performed until the filtrate was clear. The washed Fe_3_O_4_ was then placed in a muffle furnace and calcined at 300 °C for 12 hours, followed by cooling.

Step 2: a 100 g portion of the cooled Fe_3_O_4_ was weighed into a beaker, and ultrapure water was added. The Fe_3_O_4_ was then filtered out after standing for 12 hours, and the water absorption rate of the Fe_3_O_4_ was subsequently calculated.

1 Water absorption = (*m*_2_ − *m*_1_/*m*_1_ × 100%)

where: *m*_1_ represents the weight of 100 g of Fe_3_O_4_ before water absorption, and *m*_2_ is the weight of 100 g of Fe_3_O_4_ after water absorption.

Step 3: the precursors Mn(NO_3_)_2_ and Cu(NO_3_)_2_·3H_2_O were loaded onto Fe_3_O_4_ using the equal-volume impregnation method. The precursor solution was first mixed homogeneously in a beaker. Fe_3_O_4_ was then placed into another beaker, and the precursor solution was poured into the beaker while stirring. The solution was impregnated at room temperature for 12 hours, with continuous stirring to ensure uniform impregnation of Fe_3_O_4_. Once impregnation was complete, the mixture was placed into a constant-temperature drying oven at 80 °C to dry, followed by calcination in a muffle furnace at 300 °C for 12 hours. The catalyst was then cooled, washed, and dried with ultrapure water for further use.

**Fig. 1:**
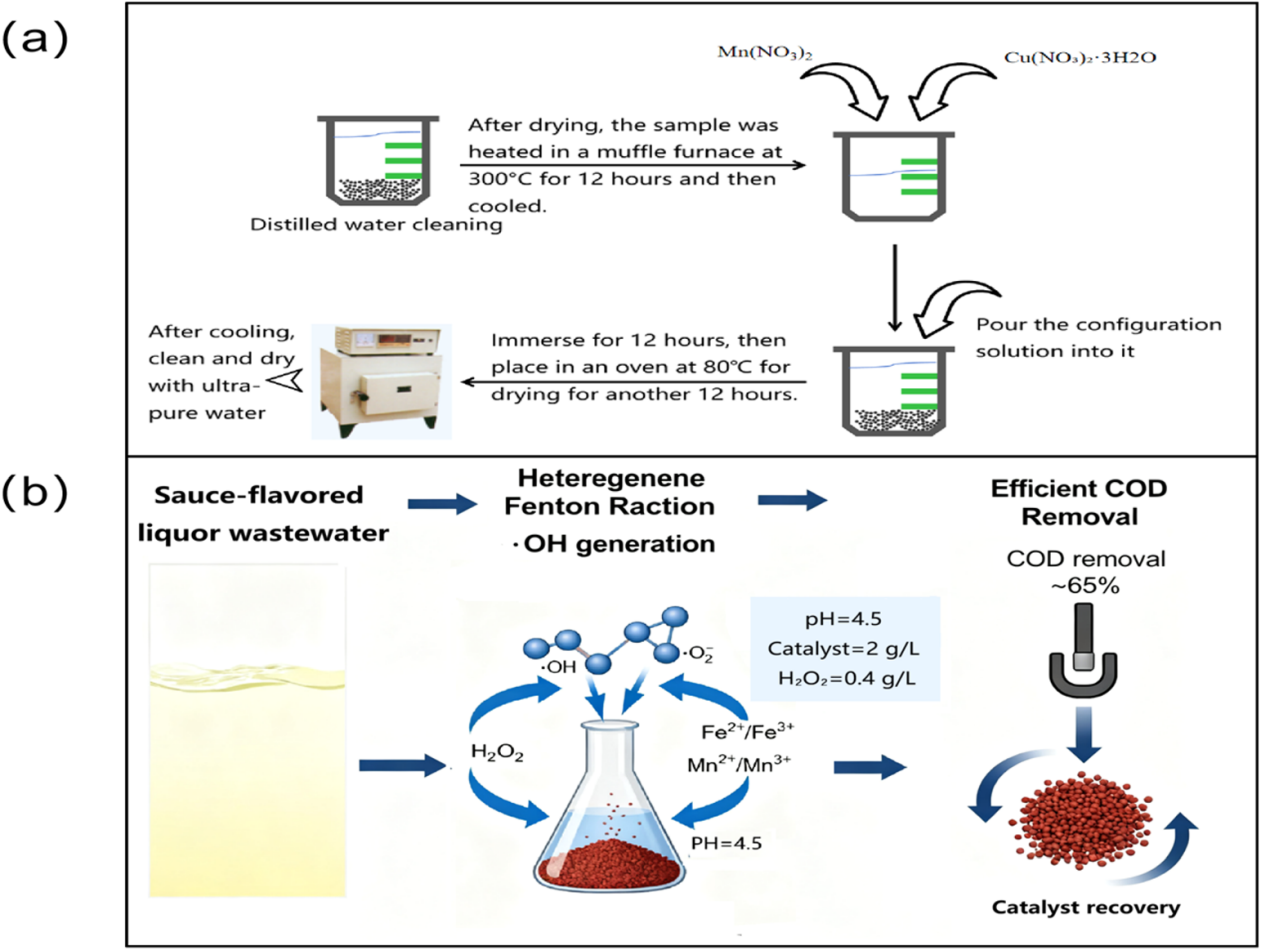
(a) Catalyst preparation process. (b) Schematic diagram of the process of pollutant removal and magnetic recovery.

In this experiment, nine catalysts with different loading ratios were prepared following the steps outlined above, as shown in Table 1. The process of catalyst preparation and pollutant removal is shown in Fig. 1. The most effective metal oxide catalysts and their optimal loading ratios for heterogeneous phase catalytic oxidation were compared and selected.

**Table 1:** Distribution ratio of different catalyst impregnation solutions

Number	Mn(NO_3_)_2_	Cu(NO_3_)_2_·3H_2_O
1	1 wt%	
2	2 wt%	
3	3 wt%	
4		1 wt%
5		2 wt%
6		3 wt%
7	1 wt%	1 wt%
8	2 wt%	1 wt%
9	3 wt%	1 wt%

### 2.3 Catalyst recovery

After the reaction was terminated, the reaction mixture was transferred to a beaker. A magnet was placed firmly against the outer wall of the beaker, and the catalyst was allowed to adsorb for 10 minutes. Once the magnetic catalyst had completely settled, the supernatant was removed stepwise by decantation. The catalyst was then collected using magnetic separation and washed with deionized water under magnetic stirring to remove any surface residues. Finally, the purified catalyst was placed in a vacuum drying oven and processed at a constant temperature to obtain a dry magnetic catalyst suitable for recycling.

## 3. Results and discussion

### 3.1 Oxidation performance test of synthesized catalysts

#### 3.1.1 Effect of type of synthesized catalyst on treatment effectiveness

Four 500 mL measuring cylinders were prepared. Each cylinder was filled with 200 mL of water samples (COD = 40 mg L^−1^) for the reaction. Aeration devices were set up in the cylinders to achieve adequate stirring during the reaction. The pH was adjusted using dilute sulfuric acid as needed. The appropriate amount of 7.5% (w/w) H_2_O_2_ solution and catalyst were weighed for each experiment. The 7.5% (w/w) H_2_O_2_ solution was added quickly, followed by the catalyst, and the mixture was stirred thoroughly using glass rods to ensure complete mixing. Once mixed, the aeration heads were turned on, and the reaction was timed. At the designated time, samples were immediately heated to terminate the reaction. The effect of different catalysts on the reaction was examined to identify the catalyst with the most effective performance.

**Fig. 2:**
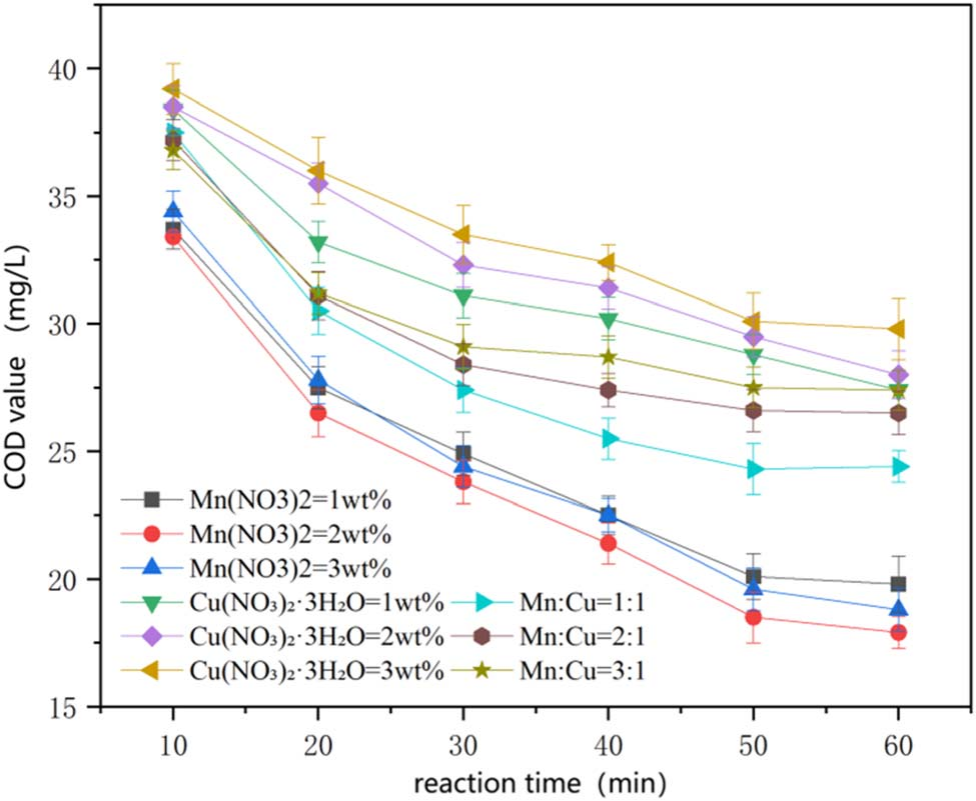
Influence of loaded catalyst type on the oxidation effect of the reaction.

As shown in Fig. 2, among the nine catalysts—Mn (wt%1, wt%2, wt%3), Cu (wt%1, wt%2, wt%3), and Mn–Cu (wt%1:wt%1, wt%2:wt%1, wt%3:wt%1)—the catalytic effect of the Mn catalysts was the best among the three categories. The post-reaction COD for the Mn catalysts reached 17.9 ± 0.62 mg L^−1^. Rational compositional tailoring of ferrite-based materials effectively modulates surface active sites and adsorption behavior, thereby strengthening interfacial processes essential for pollutant removal in advanced oxidation systems.^14^ Increasing the Mn loading from 1 wt% to 2 wt% significantly enhanced COD removal, which can be attributed to the increased density of Mn–Fe dual active sites on the Fe_3_O_4_ surface. These sites promote interfacial electron transfer and accelerate the redox cycling of Fe^2+^/Fe^3+^ and Mn^2+^/Mn^3+^, thereby facilitating the generation of reactive oxygen species, particularly ·OH. It is worth noting that increasing the loading to 3 wt% did not lead to further performance enhancement. This could be attributed to the leaching of excess Mn species from the catalyst surface during the reaction, resulting in an elevated concentration of dissolved Mn^2+^ ions. These excess ions may promote the non-productive decomposition of H_2_O_2_ or scavenge reactive oxygen species by competing with target pollutants, thereby reducing the overall oxidation efficiency. Furthermore, excessive metal loading may also cause agglomeration of active sites or pore blockage, diminishing their accessibility.

Therefore, Mn loading at 2 wt% represents an optimal balance between active site density and radical utilization efficiency, achieving high catalytic performance while avoiding radical quenching and unnecessary oxidant consumption. Considering both treatment efficiency and economic feasibility, Mn (2 wt%) was selected as the optimal loading for subsequent experiments.

#### 3.1.2 Effect of initial pH on treatment effectiveness

The optimization experiment of initial pH was conducted in two stages: first, screening the suitable pH range over a broader interval, followed by precise optimization within a narrower range. During the experiment, four 500 mL cylinders were prepared, each containing 200 mL of water samples with an initial COD of 40 mg L^−1^. An aeration device was set up to ensure sufficient mixing. Dilute sulfuric acid was used to adjust the pH of the water samples to values of 2.0, 3.0, 4.0, 5.0, 6.0, 7.0, and 8.0. The appropriate amount of 7.5% (w/w) H_2_O_2_ solution and Mn–Fe_3_O_4_ catalyst were added sequentially, after which aeration was immediately turned on. The reaction time was tracked, and the solution was stirred rapidly with a glass rod to ensure thorough mixing. Samples were taken at designated time intervals during the reaction, and immediately after removal, they were heated to terminate the reaction.

**Fig. 3:**
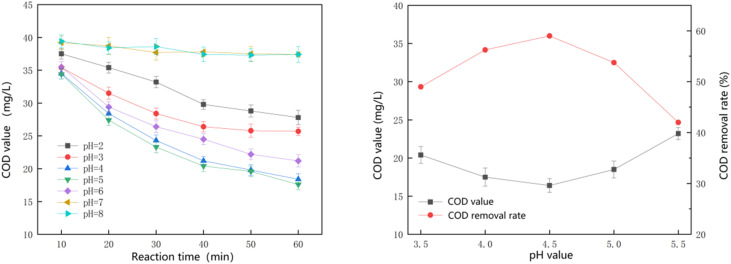
Effect of initial pH on the oxidative effect of the reaction.

Based on the results shown in Fig. 3. The catalytic system exhibited optimal performance at pH 4.5 and maintained relatively high activity within the mildly acidic range (pH 4.0–5.0). At a pH value of 4.5, the chemical oxygen demand (COD) decreased to 16.4 ± 0.9 mg L^−1^, with a corresponding COD removal rate of 59%. Although the activity decreased markedly at pH ≥ 6.0, the effective operating window is still broader than that of conventional homogeneous Fenton systems, which are typically limited to pH ≈ 3.0.

The decline in COD removal at pH ≥ 6.0 can be attributed to several interrelated factors: (i) precipitation of iron hydroxides, which reduces the availability of dissolved Fe^2+^/Fe^3+^ for Fenton reactions; (ii) accelerated non-productive decomposition of H_2_O_2_ into O_2_ and H_2_O at higher pH; and (iii) unfavorable surface charge of the catalyst, which may weaken the electrostatic adsorption of organic pollutants. These results indicate that while the Mn–Fe_3_O_4_ catalyst offers a broader working pH range than conventional Fenton, its application is optimally confined to weakly acidic conditions.

#### 3.1.3 Effect of catalyst dosage on treatment effectiveness

Four 500 mL measuring cylinders were prepared, and 200 mL of water samples (COD = 40 mg L^−1^) were added to each cylinder for the reaction. An aeration device was set up in each cylinder to achieve sufficient mixing. The pH was adjusted to 4.5 using dilute sulfuric acid. For each experiment, 0.4 g L^−1^ of 7.5% (w/w) H_2_O_2_ solution and the appropriate amount of Mn–Fe_3_O_4_ catalyst (with dosages of 1 g L^−1^, 2 g L^−1^, 3 g L^−1^, 4 g L^−1^, and 5 g L^−1^) were weighed. The catalyst was added immediately after the 7.5% (w/w) H_2_O_2_ solution, and the mixture was stirred thoroughly with a glass rod to ensure complete mixing. The aeration heads were then turned on, and the reaction was timed for 60 minutes. A sample was taken after 60 minutes, and the reaction in the sample was immediately terminated by heating.

**Fig. 4:**
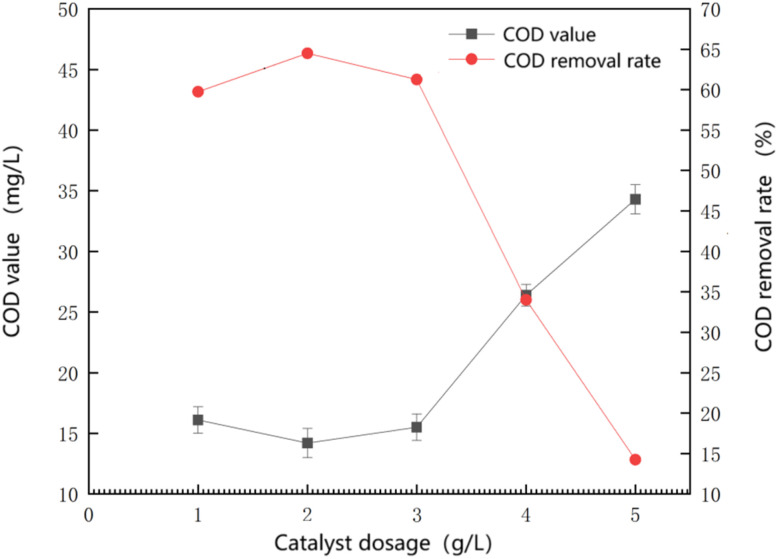
Influence of catalyst dosage on the oxidative effect of the reaction.

As shown in Fig. 4, when the dosage of the Mn–Fe_3_O_4_ catalyst is within a certain range, an increase in dosage results in a higher COD removal effect, although the fluctuation range is not large. The best removal effect was observed at a dosage of 2 g L^−1^ (0.4 g), where the COD value reached 14.2 ± 1.2 mg L^−1^, corresponding to a removal rate of 64.5%. However, when the catalyst dosage exceeds a certain threshold, the removal effect decreases linearly.

This decrease in performance at high catalyst dosages may be attributed to several concurrent factors. One possible explanation is that excessive Mn species released into the aqueous phase may accelerate the Fe^2+^/Fe^3+^ redox cycle, leading to rapid and non-productive decomposition of H_2_O_2_ and increased radical recombination. As a result, the effective concentration of ·OH available for organic oxidation may be reduced. In addition, high catalyst loadings may induce mass transfer limitations, such as particle aggregation or diffusion resistance, which can restrict the contact between reactive radicals and target pollutants. Moreover, excessive catalyst surfaces may promote the self-quenching of radicals or the scavenging of ·OH by surplus metal species, further decreasing oxidation efficiency. Therefore, the observed decline in COD removal at elevated catalyst dosages is likely the combined result of radical utilization inefficiency, mass transfer constraints, and non-productive H_2_O_2_ consumption.

#### 3.1.4 Catalyst stability tests

To evaluate the stability of the catalyst under optimal conditions, 2 g of Mn–Fe_3_O_4_ catalyst was added to 1 L of wastewater with an initial COD value of 40 mg L^−1^. A 0.4 g L^−1^ dose of 7.5% (w/w) hydrogen peroxide was added, and the reaction pH was adjusted to 4.5. Continuous aeration was maintained at the bottom of the beaker. After the reaction had proceeded for 60 minutes, the supernatant was collected, centrifuged, and digested. The COD value was measured using the potassium dichromate method. After sampling, the catalyst was fixed at the bottom of the beaker using a magnetic device. The water sample was removed, while retaining the original catalyst. A fresh batch of wastewater was added, and the reaction continued to test the catalyst's effectiveness in removing COD from sauce-flavored liquor wastewater in subsequent cycles of continuous reaction.

**Fig. 5:**
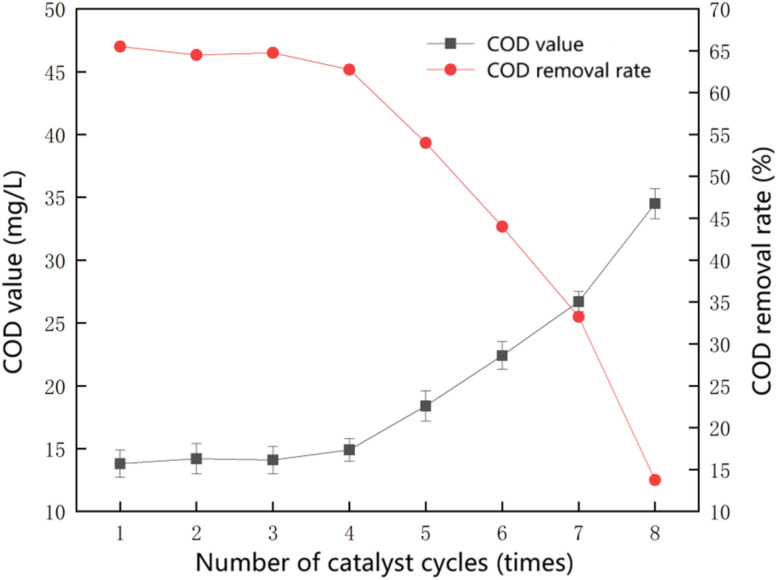
Catalyst stability experiment.

As shown in Fig. 5, the catalyst demonstrated stable performance during the first four cycles, with the effluent COD remaining below 15 mg L^−1^. However, as the number of cycles increased, the treatment efficiency of the heterogeneous Fenton reaction gradually declined. Despite this, the removal rate stabilized at 60% and above. The observed performance decrease after multiple cycles may be attributed to several possible deactivation mechanisms. The slow leaching of Mn and Fe species from the catalyst surface during reaction, leading to a reduction in the density of active redox centers; the accumulation of complex organics from wastewater or reaction intermediates, causing surface fouling or pore blockage; Furthermore, changes in surface oxidation states of Mn and Fe during prolonged redox cycling may alter electron transfer efficiency, thereby affecting hydroxyl radical generation. These results indicate that the synthesized Mn–Fe_3_O_4_ catalyst remained stable and effective in degrading COD during the advanced treatment stage of sauce-flavored liquor wastewater, highlighting its practical value as a catalyst.

### 3.2 Catalyst characterization

The prepared Mn–Fe_3_O_4_ catalysts were analyzed using SEM, XPS, EDS, and XRD to investigate changes in the morphology and microstructure of the samples after the active components were loaded onto the Mn–Fe_3_O_4_ catalysts. Additionally, the elemental compositions and valence states of the samples were analyzed.

**Fig. 6:**
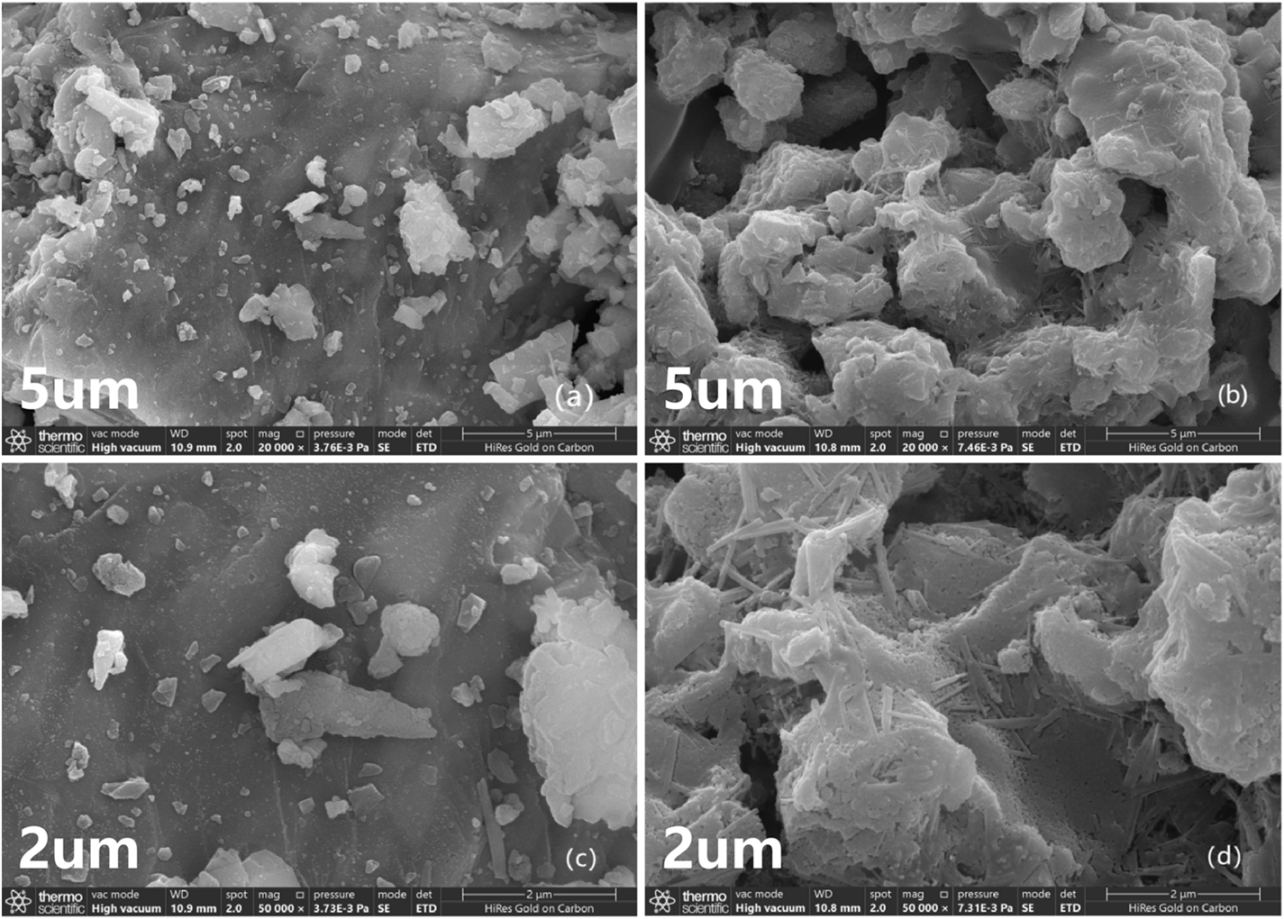
SEM images of catalysts: (a) (c): Fe_3_O_4_; (b) (d): Mn–Fe_3_O_4_.

SEM image analysis in Fig. 6 reveals that, after loading the active components (images b and d), the catalyst surface exhibits significant differences compared to the unloaded catalyst surface (images a and c). The surface of the unloaded catalyst is smoother, while the surface of the catalyst with the loaded active components shows unevenness with particles of varying sizes. This indicates successful loading of the active components onto the catalyst surface. Some active components filled the depressions on the Fe_3_O_4_ carrier surface, while others penetrated into the interior of the Fe_3_O_4_ particles. Despite this, a large number of active components still presented a raised structure on the surface of the catalyst, which contributed to the improved stability of the catalyst.

**Fig. 7:**
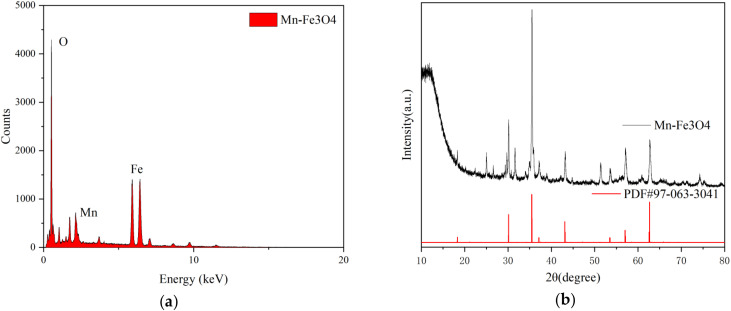
(a) Catalyst EDS image. (b) Catalyst XRD image.

Since the catalyst is magnetic, using the surface scanning method in EDS would interfere with the test results. Therefore, the test was conducted using the spot scanning method. As shown in Fig. 7, the chemical composition of the sample was determined to be Fe, O, and Mn, which confirms that the active components were successfully loaded onto the catalyst.

To verify whether the original chemical composition and structure of the catalyst were altered after treatments such as calcination, the sample was analyzed by X-ray diffraction (XRD) using an X-ray diffractometer. The test data were then compared with the PDF standard cards using JADE9. As shown in Fig. 7(b), compared with pristine Fe_3_O_4_, the diffraction peaks of Mn–Fe_3_O_4_ exhibit a slight shift toward lower 2*θ* values. According to Bragg's law, a decrease in diffraction angle corresponds to an increase in interplanar spacing (*d*-spacing), indicating lattice expansion. This lattice expansion suggests that Mn ions were successfully incorporated into the Fe_3_O_4_ spinel lattice. The larger ionic radius of Mn^2+^ (0.83 Å) compared to Fe^3+^ (0.65 Å) or Fe^2+^ (0.78 Å) may contribute to the enlargement of lattice parameters when Mn partially substitutes Fe in tetrahedral or octahedral sites. Such ionic substitution induces structural distortion and internal strain, resulting in peak displacement toward lower angles. The absence of additional diffraction peaks further confirms that Mn species are incorporated into the spinel framework rather than forming separate crystalline phases. The lattice expansion may modify the electronic structure and facilitate electron transfer between Fe^2+^/Fe^3+^ and Mn^2+^/Mn^3+^ redox couples, thereby enhancing catalytic activity in heterogeneous Fenton reactions.

**Fig. 8:**
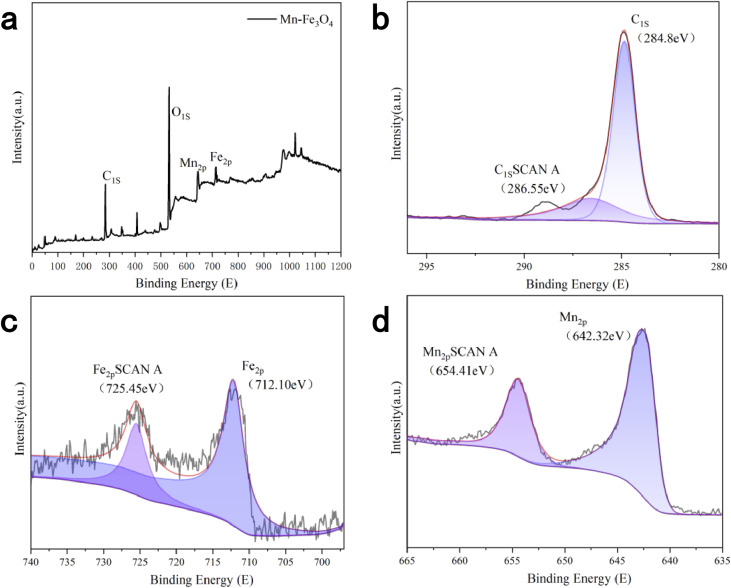
(a) XPS images of Mn–Fe_3_O_4_ catalysts (b) XPS peak-fitting images of elemental C (c) XPS peak-fitting images of elemental Fe (d) XPS peak-fitting images of elemental Mn.

To investigate the changes in chemical valence and composition of the Mn–Fe_3_O_4_ catalyst after loading the active components, XPS was used to characterize the chemical composition of the catalyst. The results, shown in Fig. 8(a), clearly identify the chemical composition of the catalyst, including Fe (2p), O (1s), Mn (2p), and C (1s) (used for correction in the analysis). The standard peak for C (1s) was observed at 284.8 eV. After correcting with C (1s), the peaks for O (1s), Fe (2p), and Mn (2p) in the Mn–Fe_3_O_4_ catalyst were located at 532.55 eV, 712.10 eV, and 642.32 eV, respectively. These characteristic peaks confirm that the active components have been successfully loaded onto the catalyst during the preparation process.

From the split-peak image of elemental C in Fig. 8(b), it can be observed that elemental C primarily exists on the catalyst surface in the form of C–C/C=O (284.8 eV). The split-peak fitting results show two peaks at 284.8 eV and 286.55 eV, respectively.^15^

From the split-peak fitting images of the elements Fe and Mn in Fig. 8(c) and (d), the peaks for Mn are located at 642.32 eV and 654.41 eV. By comparing these with standard energy spectrograms and analyzing the peak positions, it is evident that there are no significant satellite peaks in these two split peaks, and the energy difference (Δ*E*) between the two peaks is substantial. This suggests the presence of Mn^3+^ or Mn^2+^, both of which are effective in catalyzing the heterogeneous Fenton reaction;^16,17^ for the Fe element, the peaks are observed at 712.10 eV and 725.45 eV. By comparing these results with previous XRD data, it is confirmed that iron in Fe_3_O_4_ exists in both Fe^2+^ and Fe^3+^ forms, which is also reflected in the XPS peak splitting images. In XPS, iron compounds can exhibit high or low spin states. Fe^3+^ compounds typically show high spin, resulting in complex multiple split Fe_2_p spectra,^18^ while Fe^2+^ can exhibit both high and low spin states, and both forms can display satellite peaks. These satellite peaks appear between the two main peaks in the spectra;^19^ by comparing the standard spectra, it is concluded that the Fe in the catalyst exists in both divalent (Fe^2+^) and trivalent (Fe^3+^) forms, while Mn predominantly exists in its divalent (Mn^2+^) state. This provides a foundation for the subsequent mechanism analysis.

### 3.3 Kinetic analysis of heterogeneous reaction of loaded Fe_3_O_4_ catalysts

**Fig. 9:**
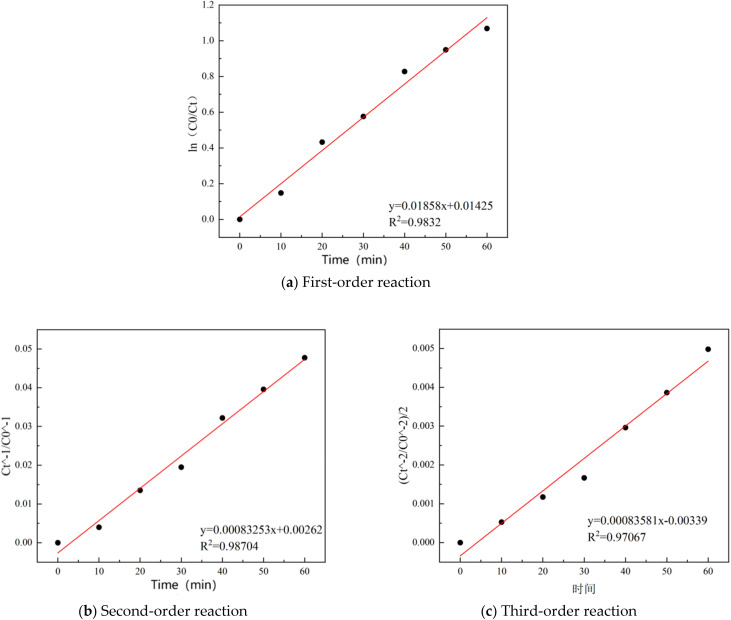
Linear regression results of the order of heterogeneous reaction.

To investigate the apparent kinetic behavior of COD degradation in sauce-flavored liquor wastewater by the synthesized catalyst. The experimental data were fitted with first-order, second-order, and third-order kinetic models.^20^ The wastewater COD is a lumped parameter contributed by a variety of organic compounds, and its degradation likely involves numerous parallel reactions. Therefore, the kinetic analysis herein aims to find an empirical mathematical model that best describes the overall degradation trend of this complex system. The obtained ‘reaction order’ thus carries an apparent meaning. The reaction kinetics are presented in Fig. 9(a)–(c).

The second-order kinetic model yields the highest goodness-of-fit (*R*^2^ = 0.98704) for our experimental data in Fig. 9. Under the optimal conditions, the apparent second-order rate constant (*k*_app) was determined to be 8.325 × 10^−4^ L mg^−1^ min^−1^. Although in complex systems such as real wastewater, the *R*^2^ values for models of different orders can sometimes be close, the principle of best fit justifies employing the second-order model as a reasonable empirical descriptor for the overall apparent behavior of COD degradation. In the field of heterogeneous catalysis, this model is often associated with a reaction rate controlled by surface processes, such as adsorption or interfacial reactions between active sites and pollutants, which is consistent with the dual-active-center mechanism proposed in this study. However, COD is a “lumped parameter” representing the total concentration of diverse organic compounds, the term “second-order kinetics” here should be understood as a best empirical approximation of the macroscopic removal trend, and the derived rate constant *k* is an apparent rate constant. Therefore, the value of this model lies in its effectiveness for quantifying and comparing the overall treatment performance of the system under different conditions, while it inherently simplifies the complex degradation network and does not define specific microscopic reaction steps.

The equation represents the concentration of reactants as a function of time, where *C*_0_ is the initial concentration of reactants, *C*_*t*_ is the concentration of reactants at time *t*, and the reaction rate constant *k* represents the rate of the reaction.

2 *C*_*t*_^−1^ − *C*_0_^−1^ = *k*_1_*t*

Under the second-order reaction kinetics model, the effects of pH value over time on the degradation rate of the reaction were investigated.

As the pH increased, the reaction apparent rate constant demonstrated an approximately linear relationship. When the pH was raised from 3.5 to 4.5, the apparent rate constant continued to rise, with the fitting *R*^2^ value reaching 0.98704 (Table 2), indicating a strong fit. However, as the pH increased beyond 4.5, the relationship deviated from linearity and showed a decreasing trend. The experimental results indicate that maintaining the reaction system within an appropriate pH range is essential for achieving enhanced COD removal performance.

**Table 2:** Fitting results of the apparent second-order rate constant *k* at different initial pH values

Initial PH	Dynamic model	*R* ^2^	*k*-app/min^−1^
3.5	*y* = 4.46 × 10^−4^*x* + 1.03 × 10^−3^	0.9508	4.46 × 10^−4^
4	*y* = 6.51 × 10^−4^*x* + 5.93 × 10^−4^	0.94975	6.51 × 10^−4^
4.5	*y* = 8.325 × 10^−4^*x* − 2.62 × 10^−3^	0.98704	8.325 × 10^−4^
5	*y* = 5.211 × 10^−4^*x* + 1.36 × 10^−3^	0.93257	5.211 × 10^−4^
5.5	*y* = 3.538 × 10^−4^*x* + 1.3 × 10^−3^	0.91968	3.538 × 10^−4^

### 3.4 Reaction mechanism study

The essence of Fenton oxidative degradation of organic matter in wastewater with catalysts lies in the process of enhancing the generation rate of hydroxyl radicals (·OH) in the presence of catalysts. This acceleration leads to the efficient oxidative removal of organic pollutants, ultimately resulting in the effective degradation of organic matter.

#### 3.4.1 GC-MS water sample detection

The water samples before and after treatment were analyzed using GC-MS, and the results are presented in Table 3. The heterogeneous Fenton technique demonstrated a stronger oxidizing effect on organic compounds such as sugars, water-soluble macromolecules, and aromatics in the wastewater. However, the reaction was less effective in treating aliphatic compounds and saturated fat carbonyl compounds. This suggests that the heterogeneous Fenton reaction mechanism aligns with the ·OH theory. The primary oxidizing species in the Fenton reaction are hydroxyl radicals (·OH) and superoxide anion radicals (·O_2_^−^), and the oxidation of complex organics by hydroxyl radicals depends on the generation of these radicals, which initiate a series of radical reactions.^21^

**Table 3:** Analysis of GC-MS results of water samples before and after heterogeneous Fenton reaction

Organic substance	Percentage (%)	Estimated concentration (mg L^−1^)	Molecular formula	C	H	O	Cl		S	N	P	I	Si	Br	Molecular mass	COD conversion factor
**(a) GC-MS results of water samples before reaction**																
Methoxyphenyl oxime	8.14%	3.39	C_8_H_9_NO_2_	8	9	2				1					151.2	1.96
Tetramethyl cycloheptasiloxane	4.87%	2.03	C_14_H_42_O_7_Si_7_	14	42	7							7		519.1	1.29
*o*-Phenylphenol	11.26%	4.69	C_12_H_10_O	12	10	1									170.2	2.63
Hexadecamethyl cyclooctasiloxane	8.04%	3.35	C_16_H_48_O_8_Si_8_	16	48	8							8		593.2	1.29
1,2,4-Triazine-3,5(2*H*)	2.63%	1.10	C_3_H_3_N_3_O_2_	3	3	2				2					113.1	0.78
2-([1,1′-Biphenyl]-2-yloxy)	20.61%	8.59	C_14_H_14_O_2_	14	14	2									214.3	2.46
3-Methyl-1-(trimethylsiloxy)	0.78%	0.32	C_10_H_20_OSi	10	20	1							1		184.4	2.52
2-(3-Iodopropyl)-1,3-dioxolane	2.06%	0.86	C_6_H_11_IO_2_	6	11	2						1			242.1	1.02
*N*-(Methoxymethyl)-1,1-diphenyl-*N*-[(trimethylsilyl)methyl]methylamine	0.93%	0.39	C_19_H_27_NOSi	19	27	1				1			1		313.5	2.58
*o*-Chlorobromobenzene	2.54%	1.06	C_4_H_6_BrC_1_	4	6		1							1	191.5	0.92
1-Acenaphthenol	34.96%	14.57	C_12_H_10_O	12	10	1									170.2	2.63
Triphenylphosphine oxide	0.73%	0.30	C_18_H_15_OP	18	15	1					1				278.3	2.44
3,5-Dimethylphenyl terephthalate	1.40%	0.58	C_18_H_18_O_4_	18	18	4									298.3	2.20
Ethyl 1,3-dithiane-2-carboxylate	1.06%	0.44	C_7_H_12_O_2_S_2_	7	12	2		2							192.3	1.50
Summation		41.67														

**(b) GC-MS results of water samples after reaction**																
Methoxyphenyl oxime	0.88	0.13	C_8_H_9_NO_2_	8	9	2				1					151.2	1.96
Tetramethyl cycloheptasiloxane	1.53	0.34	C_14_H_42_O_7_Si_7_	14	42	7							7		519.1	1.29
*o*-Phenylphenol	5.12	0.56	C_12_H_10_O	12	10	1									170.2	2.63
Hexadecamethyl cyclooctasiloxane	0.52	0.12	C_16_H_48_O_8_Si_8_	16	48	8							8		593.2	1.29
1,2,4-Triazine-3,5(2*H*)	0.98	0.36	C_3_H_3_N_3_O_2_	3	3	2				2					113.1	0.78
2-([1,1′-Biphenyl]-2-yloxy)	44.15	6.19	C_14_H_14_O_2_	14	14	2									214.3	2.46
3-Methyl-1-(trimethylsiloxy)	0.94	0.11	C_10_H_20_OSi	10	20	1							1		184.4	2.52
2-(3-Iodopropyl)-1,3-dioxolane	1.01	0.29	C_6_H_11_IO_2_	6	11	2						1			242.1	1.02
*N*-(Methoxymethyl)-1,1-diphenyl-*N*-[(trimethylsilyl)methyl]methylamine	1.15	0.13	C_19_H_27_NOSi	19	27	1				1			1		313.5	2.58
*o*-Chlorobromobenzene	1.12	0.35	C_4_H_6_BrC_1_	4	6		1							1	191.5	0.92
1-Acenaphthenol	35.01	4.86	C_12_H_10_O	12	10	1									170.2	2.63
Triphenylphosphine oxide	0.85	0.10	C_18_H_15_OP	18	15	1					1				278.3	2.44
3,5-Dimethylphenyl terephthalate	1.47	0.19	C_18_H_18_O_4_	18	18	4									298.3	2.20
Ethyl 1,3-dithiane-2-carboxylate	0.76	0.15	C_7_H_12_O_2_S_2_	7	12	2			2						192.3	1.50
Summation		13.89														

The GC-MS results showed that the proportion of organic matter in the sauce-flavored liquor wastewater was 93.8%, with 14 organic components detected. The substance with the highest proportion in both the untreated and treated water samples was “2-([1,1′-biphenyl]-2-yloxy)ethanol.” All other organic substances, except for this one, were reduced to lower concentrations after treatment. This indicates that the heterogeneous Fenton reaction effectively degraded most of the organic substances in the sauce-flavored liquor wastewater. The analysis results of the treated water sample showed that the amount of residual organic matter that was difficult to remove was approximately 13 mg L^−1^. The high proportion of “2-([1,1′-biphenyl]-2-yloxy)ethanol” may be attributed to the fact that the water samples in this study were wine wastewater, which contains a significant amount of alcohols. Moreover, the persistence of this compound helps explain the presence of residual COD after the heterogeneous Fenton reaction. Aromatic ether structures with biphenyl moieties are known to exhibit relatively high chemical stability and resistance to complete mineralization by hydroxyl radicals. As a result, while the Mn–Fe_3_O_4_ catalytic system effectively degraded the majority of readily oxidizable organic matter, a limited fraction of refractory compounds contributed disproportionately to the remaining COD. Therefore, the GC-MS results are consistent with the observed COD removal trend and further confirm that the residual COD originates mainly from a small number of persistent organic species rather than incomplete overall oxidation. Additionally, the oxidative degradation pathway of this compound by the heterogeneous Fenton technique could be explored in more depth, allowing for more targeted treatment strategies for sauce-flavored liquor wastewater.

#### 3.4.2 Quenching experiments

To determine whether hydroxyl radicals play a dominant role in the reaction, TBA and BQ were added as quenchers for ·OH and ·O^2−^, respectively, to investigate the catalytic mechanism of the heterogeneous Fenton reaction process.^22,23^

*Tert*-butanol is an efficient quencher of hydroxyl radicals, and when added, it rapidly reacts with and consumes the hydroxyl radicals, thus terminating the chain reaction initiated by free radicals in the Fenton process.^11^ By adding different quenchers, the hydroxyl or superoxide radicals involved in the oxidative degradation of organic pollutants are intercepted by TBA or BQ.^26^ The substances playing a major role in the reaction can be deduced based on how TBA and BQ are dosed.

**Fig. 10:**
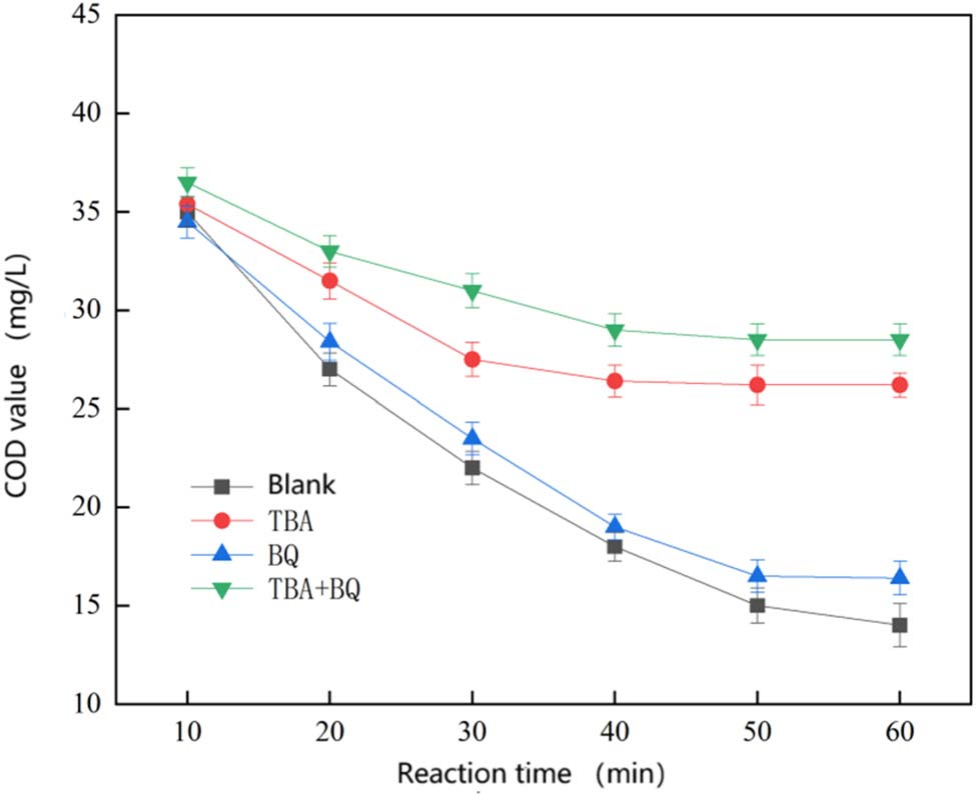
Graph of the results of the bursting experiment.

In this experiment, TBA was dosed at 10 mmol L^−1^ and BQ at 10 mmol L^−1^. The effect of free radicals on the catalytic oxidation performance of ozone was assessed by comparing the COD removal rate of each control group. The results are shown in Fig. 10.

The analysis of Fig. 10 shows that the degradation effect of the heterogeneous Fenton reaction on COD in sauce-flavored liquor wastewater was significantly reduced in the experimental group with the addition of *para*-benzoquinone and *tert*-butanol as burst agents. Among the four experimental groups, the blank control group exhibited the expected degradation effect, with the COD degradation rate reaching about 65%. In contrast, the experimental group with the addition of *tert*-butanol showed a dramatic decrease in degradation, with the COD reduced to 26.2 ± 0.6 mg L^−1^ and a degradation rate of approximately 34.5% after 60 minutes of reaction. On the other hand, the degradation effect of COD in the experimental group with *para*-benzoquinone was reduced, but the change was not significant. After the same reaction time, the COD decreased to 16.4 ± 0.8 mg L^−1^, and the degradation rate remained around 59%. Furthermore, the experimental group with both burst agents (*tert*-butanol and *para*-benzoquinone) showed the most significant reduction in reaction efficiency. After the same reaction time, the COD was reduced to only 29.5 ± 0.8 mg L^−1^, with a degradation rate of just 25%, which is about 40% lower than that of the blank control group.

A comprehensive comparison revealed that *tert*-butanol had the most significant effect on the reaction, followed by *para*-benzoquinone. After the addition of *para*-benzoquinone, the treatment effect of the reaction decreased, but the decrease was limited. This suggests that ·OH are the dominant factor in degrading organic pollutants in the heterogeneous Fenton reaction of sauce-flavored liquor wastewater with the addition of Mn–Fe_3_O_4_ catalysts, while the superoxide radical (·O_2_^−^) plays only a partial role.

#### 3.4.3 Analysis of reaction mechanisms

**Fig. 11:**
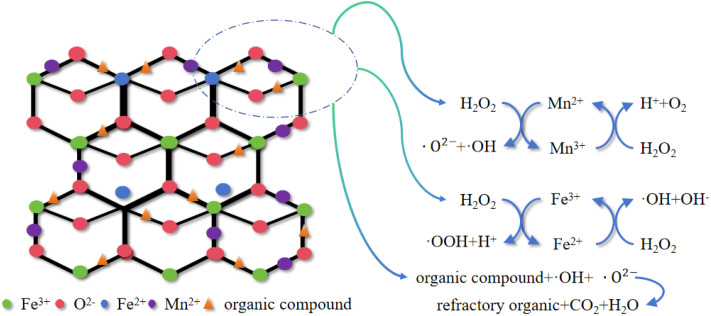
Proposed reaction mechanism diagram.

The mechanism of this heterogeneous Fenton reaction is proposed to involve enhanced free-radical generation and subsequent degradation of organic pollutants through the interaction between surface active sites of the catalyst and adsorbed pollutants. This process is likely facilitated by the synergistic effect of manganese–iron dual active centers,^24,25^ which may contribute to the improved treatment efficiency observed for sauce-flavored liquor wastewater. It has been reported that rationally designed metal-based composites facilitate reactive oxygen species production and interfacial redox cycling, offering mechanistic insights into enhanced Fenton-like catalytic oxidation.^26^ The proposed reaction mechanism is illustrated in Fig. 11, and the possible reaction process is as follows:

Organic pollutants are suggested to be initially adsorbed onto the catalyst surface, where surface active components may participate in a series of key reactions:

3 Fe^3+^ + H_2_O_2_ → Fe^2+^ + ·OOH + H^+^ (*E*° ≈ +1.09 V)

4 Mn^2+^ + H_2_O_2_ → Mn^3+^ + ·O^2−^ + ·OH (*E*° ≈ +1.13 V)

5 Fe^2+^ + H_2_O_2_ → Fe^3+^ + ·OH + OH^−^ (*E*° ≈ +0.02 V)

Synergistic effect of Fe^2+^ and Mn^3+^:Fe^2+^ generated from the above reactions is proposed to interact with Mn^3+^, while Fe^2+^ may also react with hydrogen peroxide, thereby contributing to the generation of ·OH in the heterogeneous Fenton process.^27^

6 Mn^3+^ + H_2_O_2_ → Mn^2+^ + H^+^ + O_2_ (*E*° ≈ +0.68 V)

Mn and Fe species in the system are proposed to form manganese–iron dual active centers. These dual active centers may facilitate electron transfer through charge redistribution, thereby enhancing the redox cycling between different valence states of Mn and Fe. Consequently, the generation of hydroxyl radicals is likely promoted, which may contribute to the accelerated degradation of organic pollutants.

7 ·OOH → H^+^ + ·OH + OH^−^

8 Mn^2+^ + Fe^3+^ → Mn^3+^ + Fe^2+^

XPS analysis confirmed the coexistence of Mn^2+^/Mn^3+^ and Fe^2+^/Fe^3+^ species, indicating the formation of a dual redox system. In heterogeneous Fenton reactions, Fe^2+^ reacts with H_2_O_2_ to generate ·OH (eqn (5)), while the regeneration of Fe^2+^ from Fe^3+^ governs the catalytic turnover rate. The standard redox potential of the Mn^2+^/Mn^3+^ couple (+1.51 V) is significantly higher than that of the Fe^2+^/Fe^3+^ couple (+0.77 V). Mn^2+^ can be oxidized to Mn^3+^, and this oxidation process promotes the reduction of Fe^3+^ to Fe^2+^. The continuous regeneration of Fe^2+^ facilitates the continuous generation of ·OH (+2.80 V), thereby enhancing the oxidation degradation performance. Therefore, Mn functions not only as an additional active site but also as an efficient electron shuttle that strengthens the Fe^2+^/Fe^3+^ redox loop. This synergistic Mn–Fe interaction improves electron transfer kinetics and ensures continuous reactive oxygen species generation, ultimately leading to enhanced degradation efficiency.

9 Organic compound + ·OH + ·O^2−^ → refractory organic + CO_2_ + H_2_O

The generated hydroxyl radicals (·OH) and superoxide radicals (·O_2_^−^) are suggested to participate in the oxidation of organic matter adsorbed on the catalyst surface, leading to its partial or progressive mineralization into CO_2_, H_2_O, small-molecule inorganic acids, and other oxidation products.^28,29^ As a result, a significant reduction in COD is observed in the wastewater.

## 4. Conclusions

To address the limitations of the traditional Fenton method, such as its narrow pH range, the generation of iron sludge, and the difficulty of catalyst recycling, this study proposes the use of a Mn–Fe_3_O_4_ catalyst prepared by the impregnation method for the catalytic oxidation of sauce-flavored liquor wastewater. Traditional heterogeneous Fenton oxidation catalysts are often too expensive and not suitable for the in-depth treatment of sauce-flavored liquor wastewater. The results showed that under optimal experimental conditions—2 g L^−1^ catalyst dosage, 0.4 g L^−1^ 7.5% (w/w) hydrogen peroxide dosage, pH = 4.5, and continuous aeration at the bottom—the catalyst effectively removed COD from the wastewater with good stability. Kinetic analysis of the synthesized Mn–Fe_3_O_4_ heterogeneous Fenton reaction indicated that it is more suitable for second-order reaction kinetics. The chemical composition and structural characteristics of the Mn–Fe_3_O_4_ catalyst were analyzed using SEM, XRD, XPS, and EDS. Additionally, the reaction mechanism of this catalyst for degrading organic pollutants in sauce-flavored liquor wastewater was deduced through GC-MS detection and bursting experiments. The manganese and iron elements in the reaction system exhibited good catalytic activity and a synergistic effect. The manganese–iron dual active center formed by these elements plays a crucial role in improving catalytic oxidation efficiency. The synthesized Mn–Fe_3_O_4_ catalyst offers an effective solution for the deep treatment of sauce-flavored liquor wastewater. It provides new insights into the development of heterogeneous Fenton oxidation technology and lays the foundation for future industrial applications.

## Author contributions

Luo Benfu: conceptualization, methodology, resources, writing—review and editing. Yu Jie: formal analysis, investigation, data curation, writing—original draft preparation. Yan Yujing: methodology, formal analysis, data curation. Huang Weiwei: resources, project administration. Li Jinyin: project administration, funding acquisition. Liu Yuhang: investigation, visualization. Yang Xi: software, validation. Zhou Xiang: funding acquisition. Ning Haiyan: visualization, supervision. All authors have read and agreed to the published version of the manuscript.

## Conflicts of interest

Authors Jinyin Li and Weiwei Huang were employed by the company China Municipal Engineering Zhongnan Design and Research Institute Co., Ltd. Author Xiang Zhou was employed by the company Suyi Design Group Co., Ltd. The remaining authors declare that the research was conducted in the absence of any commercial or financial relationships that could be construed as a potential conflict of interest.

## Data Availability

All data generated or analysed during this study are included in this published article.
